# Lipoprotein(a) Modulates Carotid Atherosclerosis in Metabolic Syndrome

**DOI:** 10.3389/fmolb.2022.854624

**Published:** 2022-06-08

**Authors:** Anna Laura Cremonini, Andrea Pasta, Federico Carbone, Luca Visconti, Matteo Casula, Edoardo Elia, Aldo Bonaventura, Luca Liberale, Maria Bertolotto, Nathan Artom, Silvia Minetti, Paola Contini, Daniela Verzola, Roberto Pontremoli, Francesca Viazzi, Giorgio Luciano Viviani, Stefano Bertolini, Aldo Pende, Fabrizio Montecucco, Livia Pisciotta

**Affiliations:** ^1^ Department of Internal Medicine, University of Genoa, Genoa, Italy; ^2^ IRCCS Ospedale Policlinico San Martino, Genoa, Italy; ^3^ Internal Medicine Department Ospedale di Circolo e Fondazione Macchi, ASST Sette Laghi, Varese, Italy; ^4^ Department of Internal Medicine, Ospedale S. Paolo di Savona, Savona, Italy

**Keywords:** lipoprotein (a), atherosclerosis, metabolic syndrome, cardiovascular disease, ASCVD

## Abstract

**Background and Aim:** High lipoprotein(a) [Lp(a)] is a well-established cardiovascular (CV) risk factor, but the effect of mildly elevated Lp(a) on CV health is largely unknown. Our aim was to evaluate if Lp(a) is associated with the severity of carotid atherosclerosis (CA) in the specific subset of metabolic syndrome (MetS).

**Patients and Methods:** Subjects with diagnosed MetS and ultrasound-assessed CA were enrolled. Those patients were categorized according to the severity of CA (moderate vs. severe), and the circulating levels of Lp(a) alongside with clinical, anthropometric, and biochemical data were collected.

**Results:** Sixty-five patients were finally included: twenty-five with moderate and forty with severe CA (all with asymptomatic disease). Intergroup comparison showed Lp(a) as the only significantly different variable [6 (2–12) mg/dl vs. 11.5 (6–29.5) mg/dl; *p* = 0.018]. Circulating levels of Lp(a) were also confirmed as the only variable independently associated with severity of CA at logistic regression analysis [OR 2.9 (95% CI 1.1–7.8); *p* = 0.040]. ROC curve analysis for Lp(a) confirmed a serum level of 10 mg/dl as the best cut-off value [AUC 0.675 (95% CI 0.548–0.786)]. Although sensitivity and specificity were suboptimal (69.0 and 70.4%, respectively)—likely due to the small sample size—this result is in line with those previously reported in the literature.

**Conclusion:** Lp(a) is independently associated with severity of CA in the subgroup of MetS patients.

## Introduction

When metabolic syndrome (MetS) is diagnosed (Grundy et al., 2005), patients need to be accurately evaluated because of the higher risk of developing atherosclerotic cardiovascular disease (ASCVD). Carotid intima-media thickness (C-IMT) and plaques are the surrogate marker of atherosclerosis and powerful predictor of vascular outcomes in MetS patients (Pollex et al., 2006; Rundek et al., 2007; Olmastroni et al., 2019). However, the accuracy in the stratification of the carotid atherosclerosis (CA) severity is not satisfactory using only traditional risk factors. Lipoprotein(a) [Lp(a)] is a lipoprotein consisting of a particle of apolipoprotein B linked to apolipoprotein(a) and can be considered an “emergent” risk factor for the development of ASCVD and CA (Tsimikas, 2017) due to its pro-thrombotic and pro-inflammatory effects (Orso and Schmitz, 2017), at least when markedly elevated (Emerging Risk Factors et al., 2009; Kamstrup et al., 2009). Nevertheless, Lp(a) is not routinely measured in a real-world setting, at least in part because of the lack of standardization of the dosage methods and the absence of commercially available Lp(a)-lowering therapies. Anyway, it has been postulated that Lp(a) could be a useful tool in clinical practice for identifying patients in which the atherosclerotic process is more advanced (Ezhov et al., 2014; Rigamonti et al., 2018). Furthermore, second-generation anti-sense oligonucleotides designed to target and bind to apo(a) messenger RNA (mRNA) in hepatocytes are increasingly approaching clinical practice (Katzmann et al., 2020;Lp(a)HORIZON, 2022). Characterizing the role of Lp(a) in different classes of patients at cardiovascular risk is becoming an urgent need. Here, we focused on MetS, an enhancer of CA development (Cuspidi et al., 2018). The aim of this study was the evaluation of the independent association between Lp(a) and the CA severity in a group of patients with an established diagnosis of MetS.

## Methods

### Patients

This pilot study is a sub-analysis of the previously published prospective study (Carbone et al., 2019) conducted in the outpatient clinic for the treatment of dyslipidemias and hypertension in the San Martino Hospital of Genoa, in accordance with the Declaration of Helsinki (October 2013) and approved by the Regional Ethics Committee. Informed written consent was collected from all patients at enrollment. The original cohort enrolled patients (>18 years old) with metabolic syndrome (MetS) diagnosed according to the American Heart Association (AHA) and the National Heart, Lung, and Blood Institute (NHLBI) criteria (Grundy et al., 2005). Exclusion criteria included acute coronary syndrome (unstable angina and myocardial infarction), congestive heart failure (NYHA class III-IV), abnormal liver or kidney function, acute and chronic infections (including HIV, HCV, and HBV), connective tissue diseases, solid or hematological tumors, endocrinopathies (including untreated hypothyroidism), inflammatory bowel diseases, and chronic therapy with anti-inflammatory drugs or hormonal therapy (including insulin) or with recombinant cytokines. From the original cohort, we selected patients who performed ultrasound investigation of carotid atherosclerosis and for which serum sample Lp(a) assay was available. Sixty-five patients were finally included in this cross-sectional sub-analysis.

Enrolled patients underwent complete medical examination during which anamnestic data, anthropometric values [weight, height, body mass index (BMI), and waist circumference], and vital parameters (heart rate and arterial pressure on three repeated measurements) were collected.

Venous blood was collected for the evaluation of a complete lipid profile: Total cholesterol (TC), triglycerides (TAG), and high-density lipoprotein cholesterol (HDL) levels were measured enzymatically using commercial kits by Roche. LDL cholesterol was calculated by Friedewald’s formula. Lp (a) levels were determined by a nephelometry assay (Image Immunchemie System, Beckman Coulter, Italy) with a polyclonal antibody directed against the apoprotein (a)-domain of Lp (a) in an assay insensitive to apoprotein (a) isoforms. The limit of detection was 15.62 pg/ml (Rigamonti et al., 2018).

Lp(a) was determined from the serum with nephelometric technology by means of a BN II analyzer (Gencer et al., 2019). The colorimetric enzyme-linked immunosorbent assay (R&D Systems, Minneapolis, MN) has been used for measuring the serum C-reactive protein (CRP) levels (Carbone et al., 2019).

Carotid intima-media thickness (C-IMT) at the level of the posterior wall of the distal 10 mm of the common carotid artery (CCA) and atherosclerotic lesions were assessed by echo-color Doppler technique with a 7.5-MHz linear probe and the MyLab™ Five system (Esaote Group, Genoa, Italy). The evaluation was performed by the same operator in order to minimize the inter- and intra-individual variability. CA was categorized in “moderate” (C-IMT 1.0–1.5 mm, no plaques) or “severe” (C-IMT>1.6 mm or plaques), following the international guidelines (Williams et al., 2018).

### Statistical Analysis

Statistical analysis was performed using IBM-SPSS Statistics, Release Version 25.0 (SPSS, Inc., 2017, Chicago, IL). Kolmogorov–Smirnov analysis was performed to test the normality of variables. Therefore, biomarker data were log-transformed, where necessary. Results of continuous variables were expressed as median and interquartile range (IQR). For ordinal and nominal variables, contingency tables were used, indicating frequency and percentage (%). Mann–Whitney test was then drawn for intergroup comparison of continuous variables. The primary outcome of the study was to test the independent association of Lp(a) with severity of CA. The *post hoc* study power estimated for such an outcome was 0.568 (*p* = 0.034). The present study should be then considered as a pilot.

To identify independent variables associated with severity of CA , we then performed logistic regression analysis. Variables were log-transformed, where necessary. Finally, the receiver operating characteristic (ROC) curve has been used to verify sensibility and specificity of the selected cut-off point of Lp(a) (MedCalc 12.5, MedCalc Software, Ostend, Belgium). All hypothesis tests were two-sided, and the significance level was set at 5%.

## Results

Demographic, clinical, and anthropometric data on sixty-five patients with evidence of asymptomatic CA are summarized in Table 1. All patients were Caucasian and without history of familial hypercholesterolemia, the median age was 58 years old, 61.5% of patients were male, and 29.2% were current smokers. As expected, BMI and waist circumference were high (median values were 29.4 kg/m^2^ and 105 cm, respectively), reflecting the typical accumulation of visceral adipose tissue in these kind of patients. It is worth noting that despite MetS criteria being well represented in the cohort, the median 10-year ASCVD risk was 1.8, which was considered moderate. Accordingly, only one patient had a clinical history of ASCV disease. Lp(a) values range from 1 to 192 mg/dl (Supplementary Figure S1).

**TABLE 1:** Characteristics of sixty five patients with metabolic syndrome.

Variable	Value
Age, years [IQR]	58 (51–61)
Sex, male (%)	40 (61.5)
Waist circumference, cm [IQR]	105 (100–112)
Weight, kg [IQR]	86 (74–97)
BMI, kg/m^2^ [IQR]	29.4 (27.7–32.6)
sBP, mmHg [IQR]	143 (132–152)
dBP, mmHg [IQR]	85 (80–92)
Active smoker, (%)	19 (29.2%)
T-c, mg/dl [IQR]	241 (204–267)
HDL-C, mg/dl [IQR]	37 (33–46)
Non-HDL-c, mg/dL [IQR]	191 (162–222)
LDL-C, mg/dl [IQR]	153 (117–177)
TG, mg/dl [IQR]	231 (157–340)
Glycemia, mg/dl [IQR]	104 (94–114)
HbA1c mmol/mol [IQR]	41 (36–44)
c-IMT, mm [IQR]	1.00 (.90–1.10)
Lp(a), mg/dl [IQR]	10.0 (4.0–24.0)
CRP, mg/L [IQR]	2.8 (1.7–7.0)
Ultrasound carotid plaque, n (%)	39 (60.0%)
10-year ASCVD risk (SCORE), % [IQR]	1.8 (0.9–4.2)
MetS criteria	
*n* = 3 (%)	27 (41.5%)
*n* = 4 (%)	23 (35.4%)
*n* = 5 (%)	15 (23.1%)
Statin use, n (%)	14 (21.50)
Ezetimibe use, n (%)	4 (6.2)
Aspirin use, n (%)	10 (15.4)
ACE-inhibitor use, n (%)	10 (15.4)
ARB-inhibitor use, n (%)	33 (50.8)

M, male; BMI: body mass index; IQR, interquartile range; SBP, systolic blood pressure; DBP, diastolic blood pressure; T-c, total cholesterol; LDL-c, low-density lipoprotein cholesterol; HDL-c, high-density lipoprotein cholesterol; TG, triglyceride; HbA1c, glycated hemoglobin; c-IMT, carotid intima-media thickness; Lp(a), lipoprotein (a); CRP, C-reactive protein; ASCVD, atherosclerotic cardiovascular disease; MetS, metabolic syndrome.

Among those different CV risk factors, Lp(a) emerged as the only variable differing across the study groups (moderate vs. severe CA), as reported in Table 2. Indeed, despite a prevalence of males (65% vs. 56%) and smokers (7% vs. 12%) and, generally, a worse metabolic profile, only the levels of Lp(a) were significantly different [6 (2 to 12)] mg/dl vs*.* 11.5 (6 to 29.5)] mg/dl; *p* = 0.018. We also observed no differences in the pharmacological history of patients. In this regard, no patient was in active treatment with nicotinic acid and/or PCSK9 inhibitors.

**TABLE 2:** Characteristics of 65 patients with metabolic syndrome divided according to the severity of carotid atherosclerosis identified with the echo-color Doppler study.

Carotid atherosclerosis	Moderate 0.1–1.5 mm (*n* = 25)	Severe >1.6 mm and/or plaque (*n* = 40)	*p*-value
Age, years [IQR]	56 (49–59)	59 (54–62)	0.216
Sex, male (%)	14 (56.0)	26 (65.0)	0.468
Waist circumference, cm [IQR]	104 (96–111)	107 (102–113)	0.641
Weight, kg [IQR]	82 (72–98)	86 (79–96)	0.518
BMI, kg/m^2^ [IQR]	30.3 (28.3–33.7)	29.1 (27.6–32.2)	0.434
sBP, mmHg [IQR]	145 (140–152)	143 (131–151)	0.491
dBP, mmHg [IQR]	88 (80–94)	85 (80–90)	0.457
Active smoker, (%)	7 (28.0)	12 (30.0)	0.863
Tc, mg/dL [IQR]	236 (205–257)	249 (203–276)	0.434
HDL-C, mg/dL [IQR]	40 (34–48)	37 (32–45)	0.295
Non-HDL-c, mg/dL [IQR]	171 (153–205)	196 (169–225)	0.064
LDL-C, mg/dL [IQR]	153 (125–177)	153 (109–175)	0.989
TG, mg/dL [IQR]	201 (166–264)	264 (149–363)	0.184
Glycemia, mg/dL [IQR]	101 (95–109)	106 (94–116)	0.232
HbA1c mmol/mol [IQR]	38 (35–43)	42 (37–45)	0.136
Lp(a), mg/dl [IQR]	6 (2–12)	11.5 (6–29.5)	**0.018**
CRP, mg/L [IQR]	2.87 (1.66–7.37)	2.57 (1.6–6.97)	0.976
10-year ASCVD risk (SCORE), % [IQR]	1.77 (0.63–4.17)	2.32 (0.94–4.07)	0.716
MetS criteria			0.592
*n* = 3 (%)	12 (48.0%)	15 (37.5%)	
*n* = 4 (%)	7 (28.0%)	16 (40.0%)	
*n* = 5 (%)	6 (24.0%)	9 (22.5%)	
Statin use, *n* (%)	6 (24.0)</u>	8 (20.0)	0.762
Ezetimibe use, *n* (%)	2 (8.0)	2 (5)	0.635
Aspirin use, *n* (%)	1 (4.0)	9 (22.5)	0.075
ACE-inhibitor use, *n* (%)	4 (16.0)	6 (15.0)	1.000
ARB-inhibitor use, *n* (%)	9 (36.0)	24 (60.0)	0.798

*p*-value# refers to comparisons between moderate and severe groups analyzed with the Mann–Whitney test. M, male; BMI, body mass index; IQR, interquartile range; SBP, systolic blood pressure; DBP, diastolic blood pressure; TC, total cholesterol; LDL-C, low-density lipoprotein cholesterol; HDL-C, high-density lipoprotein cholesterol; TG, triglyceride; G, glycemia; HbA1c, glycated hemoglobin; Lp(a), lipoprotein (a); CRP, C-reactive protein; ASCVD, atherosclerotic cardiovascular disease; MetS, metabolic syndrome; ACE, angiotensin-converting enzyme; ARBs, angiotensin receptor blockers.

Bold underlines significant values.

**FIGURE 1:**
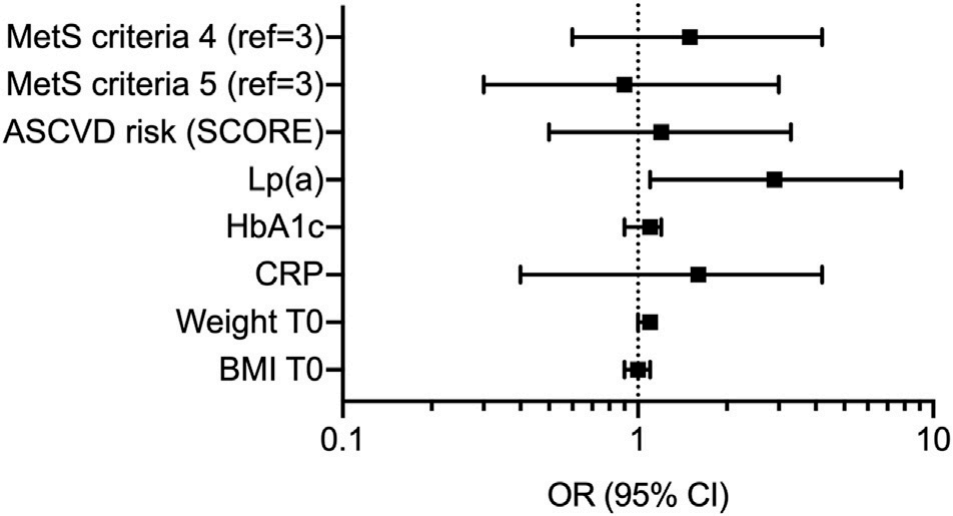
Forest plot reporting logistic regression analysis for clinical and biochemical variables associated with more severe carotid atherosclerosis. Data are presented as odds ratio (OR) with 95% confidence interval (CI). MetS, metabolic syndrome; ASCVD, atherosclerotic cardiovascular disease; Lp(a), lipoprotein(a); HbA1c, glycated hemoglobin; CRP, C-reactive protein; BMI, body mass index.

**FIGURE 2:**
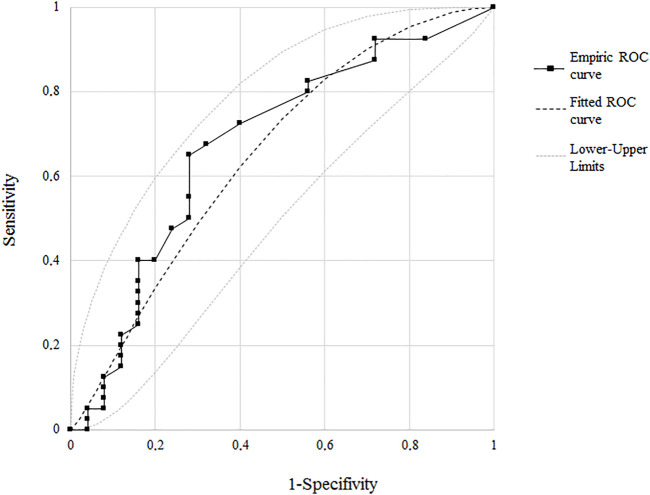
ROC curve illustrating the association between Lp(a) and the severity of carotid atherosclerosis of the cut-off value for carotid atherosclerosis. Sens, sensibility; Spec, specificity; AUC, area under the curve.

In line with those observations, logistic regression analysis confirmed serum levels of Lp(a) as the only variable independently associated with severity of CA (Figure 1 and Supplementary Table S1) The OR for Lp(a) was indeed 2.9 with a 95% CI of 1.1–7.8. When the ROC curve was performed, a cut-off of 10 mg/dl was the best Lp(a) value, identifying patients with more severe atherosclerosis with an AUC of 0.675 (95% CI 0.548–0.786), a sensitivity of 69.0%, and a specificity of 70.4% (Figure 2).

## Discussion and Conclusion

The major finding of the present study was the independent association between Lp(a) levels and the severity of asymptomatic CA in a selected group of patients with an established diagnosis of MetS. As widely known, MetS represents a cluster of metabolic disturbances mainly derived from the accumulation of visceral adipose tissue, and it is mild but with chronic inflammation (Gustafson et al., 2007). This condition is strictly related to the higher risk of atherosclerosis, and several studies have documented the higher prevalence of CA in MetS patients (Grundy et al., 2005; Pollex et al., 2006). Lp(a) is increasingly described as an emerging contributor to the development of atherosclerotic plaque. Several pieces of experimental evidence have indeed demonstrated the pathogenetic role of Lp(a) in atherosclerosis due to its prothrombotic/anti-fibrinolytic effect and/or its ability to cross the endothelial barrier and accumulate in the arterial wall (Gustafson et al., 2007). Therefore, it seems that the longer the lifetime exposure to high Lp(a) plasma concentrations, the higher is the risk of ASCVD (Clarke et al., 2009; Kamstrup et al., 2009). Our results are somehow confirmative of this hypothesis even though the overall values are lower than values elsewhere reported.

In the present study, Lp(a) was shown to be the only independent variable discriminating two subgroups of patients categorized according to the severity of atherosclerotic lesions. Moreover, we were able to identify a relatively low cut-off value (10 mg/d), which could help at identifying subjects with higher C-IMTs or atherosclerotic plaques. We may also speculate about a future role of Lp(a)—even at low circulating levels—as a useful biomarker for implementing CV risk stratification. This could be applied in both primary and secondary CV prevention, combined with other recognized risk factors (as occurring in MetS).

We should acknowledge that the present study has many limitations. First, the small sample size does not allow in drawing any conclusion about clinical relevance of Lp(a) in CV risk stratification and the proportion of outlier. However, the cut-off point identified meets which were observed in the previous larger studies (Emerging Risk Factors et al., 2009; Rigamonti et al., 2018), and this may be considered a major strength of the present study.

Second, the use of nephelometric assay for the dosage of Lp(a) did not allow in evaluating the different apoprotein(a) [apo(a)] isoforms. It is indeed well-established that the lower the molecular weight of apo(a), the higher is the Lp(a) concentration and the risk of atherosclerotic disease (Kraft et al., 1996). Conversely, there is a lack of clinical evidence on how aggressive lipoprotein(a) lowering reduction improves the following ASCVD risk reduction. Attention should be then maintained on the other modifiable CV risk factors (Ruscica et al., 2021; Melita et al., 2022). Furthermore, we should acknowledge a considerable amount of outlier—especially toward higher values—that should affect statistical analysis. We then have to reaffirm that the findings have to be considered preliminary.

In conclusions, this study reported that even relatively low circulating Lp(a) may be useful in discriminating patients with more severe CA. Many ongoing clinical trials are called to confirm our preliminary observation, and they eventually identify whether Lp(a) might have a clinical use in CV risk stratification (NCT03887520, NCT04023552, and NCT04310917). They are also expected to identify Lp(a) as a potential biomarker useful to address therapeutic strategies toward a more aggressive approach.

## Data Availability

The raw data supporting the conclusions of this article will be made available by the authors, without undue reservation.

## Supplementary Material

Supplementary Figure S1 Box plot illustrating Lp(a) distribution across the study groups. Black circles represent the outlier values as identified by the iterative Grubb’s test.

Supplementary Table S1 Regression analysis for TSA severity in patients with evidence of carotid atherosclerosis.
