# Supplementary material for: Lipoprotein(a) Modulates Carotid Atherosclerosis in Metabolic Syndrome
Source: Front Mol Biosci. 2022 Jun 8;9:854624. doi: 10.3389/fmolb.2022.854624 (PMC9214261; doi:10.3389/fmolb.2022.854624)

**Supplementary Material**

**Supplementary Table 1. Regression analysis for TSA severity in patients with evidence of carotid atherosclerosis**

|  | **Univariate** | |
| --- | --- | --- |
| **Variable** | **OR (95% CI)** | **p-value** |
| MetS criteria 4 (ref=3) | 1.5 (0.6 - 4.2) | 0.404 |
| MetS criteria 5 (ref=4) | 0.9 (0.3 – 3.0) | 0.889 |
| 10-years ASCVD risk (SCORE) | 1.2 (0.5 – 3.3) | 0.671 |
| Lp(a) | 2.9 (1.1 – 7.8) | **0.040** |
| HbA1c | 1.1 (0.9 - 1.2) | 0.112 |
| CRP | 1.6 (0.4 – 4.2) | 0.595 |
| Weight | 1.1 (1.0- 1.1) | 0.723 |
| BMI | 1.0 (0.9 – 1.1) | 0.971 |

OR: odds ratio; CI: confidence interval; MetS: metabolic syndrome; ASCVD: atherosclerotic cardiovascular disease; Lp(a): lipoprotein a; HbA1c: glycated hemoglobin; CRP: C-reactive protein; BMI: body mass index.

**Supplementary Figure 1.** Box plot illustrating Lp(a) distribution across the study groups. Black circles represent the outlier values as identified by the iterative Grubb’s test.


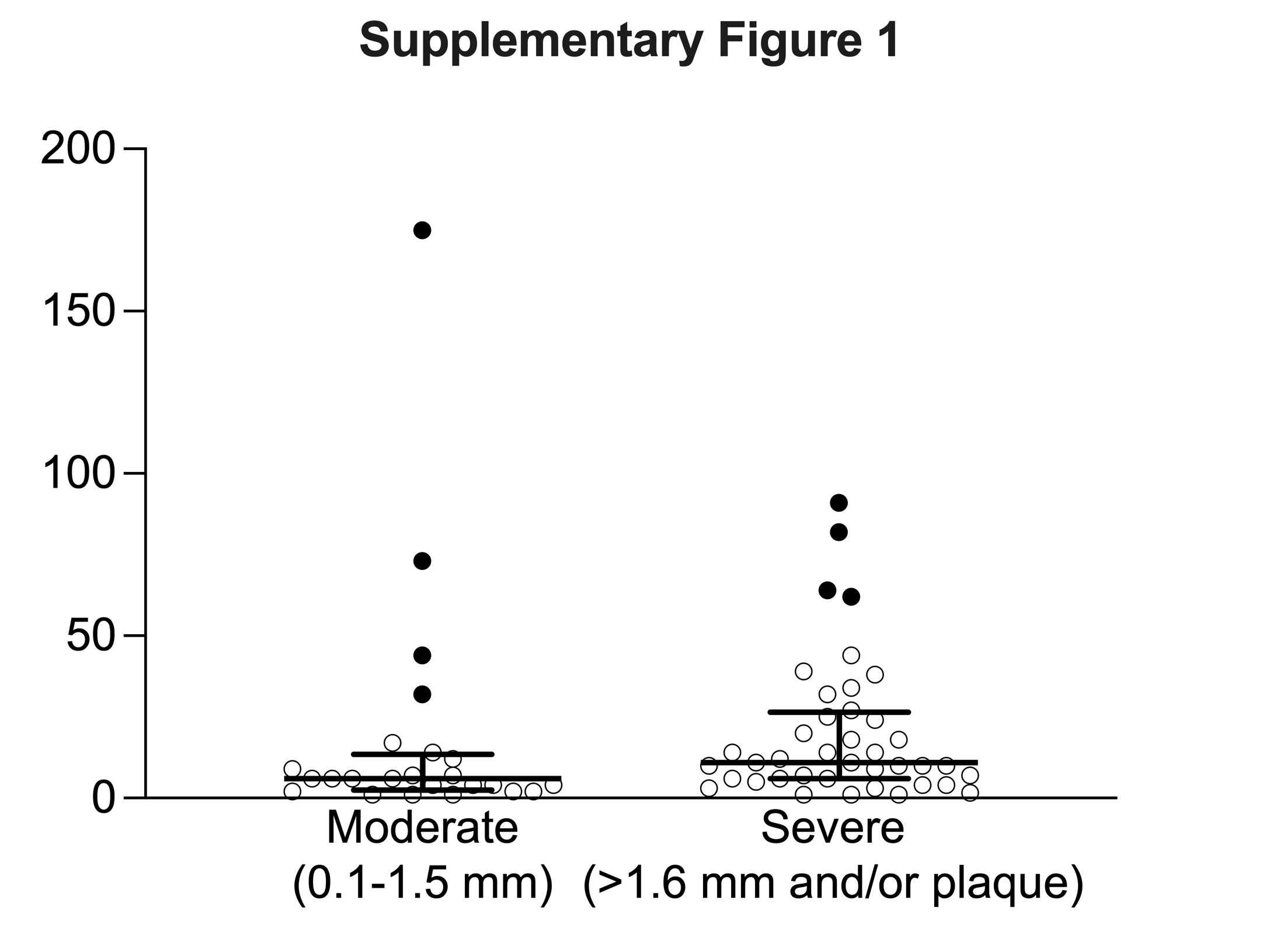

Supplement: Supplementary file 1 [file Table1.DOCX]
